# Psychometric Assessment of an Item Bank for Adaptive Testing on Patient-Reported Experience of Care Environment for Severe Mental Illness: Validation Study

**DOI:** 10.2196/49916

**Published:** 2024-05-16

**Authors:** Sara Fernandes, Yann Brousse, Xavier Zendjidjian, Delphine Cano, Jérémie Riedberger, Pierre-Michel Llorca, Ludovic Samalin, Daniel Dassa, Christian Trichard, Vincent Laprevote, Anne Sauvaget, Mocrane Abbar, David Misdrahi, Fabrice Berna, Christophe Lancon, Nathalie Coulon, Wissam El-Hage, Pierre-Emmanuel Rozier, Michel Benoit, Bruno Giordana, Alejandra Caqueo-Urízar, Dong Keon Yon, Bach Tran, Pascal Auquier, Guillaume Fond, Laurent Boyer

**Affiliations:** 1 Assistance Publique-Hopitaux de Marseille, Aix-Marseille University, UR3279: Health Service Research and Quality of Life Center - CEReSS Marseille France; 2 Department of Psychiatry, Centre Hospitalier Universitaire de Clermont-Ferrand, University of Clermont Auvergne, Centre national de la recherche scientifique, Institut national polytechnique de Clermont Auvergne, Institut Pascal UMR 6602 Clermont-Ferrand France; 3 Etablissement public de santé Barthélemy Durand Etampes France; 4 Department of Addictology and Psychiatry, Centre Psychothérapique de Nancy Laxou France; 5 Département de Psychiatrie, Centre Hospitalier Régional Universitaire de Strasbourg, Université de Strasbourg, Institut national de la santé et de la recherche médicale U1114, Fédération de Médecine Translationnelle de Strasbourg Strasbourg France; 6 Nantes Université, Centre Hospitalier Régional Universitaire de Nantes, Movement - Interactions - Performance - MIP UR 4334 Nantes France; 7 Department of Psychiatry, Centre Hospitalier Universitaire de Nîmes, University of Montpellier Nîmes France; 8 National Centre for Scientific Research UMR 5287 - Institut de Neurosciences Cognitives et Intégratives d'Aquitaine, University of Bordeaux, Centre Hospitalier Charles Perrens Bordeaux France; 9 Centre Expert Schizophrénie, Centre Expert TSA-SDI et Centre Référent de Réhabilitation Psychosociale et de Remédiation Cognitive - C3R, Centre Hospitalier Alpes Isère Grenoble France; 10 Centre Hospitalier Régional Universitaire de Tours, Clinique Psychiatrique Universitaire Tours France; 11 Department of Psychiatry, Centre Hospitalier des Pyrénées Pau France; 12 Department of Psychiatry, Hopital Pasteur, University Hospital of Nice Nice France; 13 Instituto de Alta Investigación, Universidad de Tarapacá Arica Chile; 14 Center for Digital Health, Medical Science Research Institute, Kyung Hee University College of Medicine, Department of Pediatrics, Kyung Hee University Medical Center Seoul Republic of Korea; 15 Institute of Preventive Medicine and Public Health, Hanoi Medical University Hanoi Vietnam

**Keywords:** psychiatry, public mental health, schizophrenia, major depressive disorders, bipolar disorders, patient-reported experience measures, quality of care, health services research, computerized adaptive testing, real-world data

## Abstract

**Background:**

The care environment significantly influences the experiences of patients with severe mental illness and the quality of their care. While a welcoming and stimulating environment enhances patient satisfaction and health outcomes, psychiatric facilities often prioritize staff workflow over patient needs. Addressing these challenges is crucial to improving patient experiences and outcomes in mental health care.

**Objective:**

This study is part of the Patient-Reported Experience Measure for Improving Quality of Care in Mental Health (PREMIUM) project and aims to establish an item bank (PREMIUM-CE) and to develop computerized adaptive tests (CATs) to measure the experience of the care environment of adult patients with schizophrenia, bipolar disorder, or major depressive disorder.

**Methods:**

We performed psychometric analyses including assessments of item response theory (IRT) model assumptions, IRT model fit, differential item functioning (DIF), item bank validity, and CAT simulations.

**Results:**

In this multicenter cross-sectional study, 498 patients were recruited from outpatient and inpatient settings. The final PREMIUM-CE 13-item bank was sufficiently unidimensional (root mean square error of approximation=0.082, 95% CI 0.067-0.097; comparative fit index=0.974; Tucker-Lewis index=0.968) and showed an adequate fit to the IRT model (infit mean square statistic ranging between 0.7 and 1.0). DIF analysis revealed no item biases according to gender, health care settings, diagnosis, or mode of study participation. PREMIUM-CE scores correlated strongly with satisfaction measures (*r*=0.69-0.78; *P*<.001) and weakly with quality-of-life measures (*r*=0.11-0.21; *P*<.001). CAT simulations showed a strong correlation (*r*=0.98) between CAT scores and those of the full item bank, and around 79.5% (396/498) of the participants obtained a reliable score with the administration of an average of 7 items.

**Conclusions:**

The PREMIUM-CE item bank and its CAT version have shown excellent psychometric properties, making them reliable measures for evaluating the patient experience of the care environment among adults with severe mental illness in both outpatient and inpatient settings. These measures are a valuable addition to the existing landscape of patient experience assessment, capturing what truly matters to patients and enhancing the understanding of their care experiences.

**Trial Registration:**

ClinicalTrials.gov NCT02491866; https://clinicaltrials.gov/study/NCT02491866

## Introduction

The health care environment, which encompasses design features (ie, cleanliness, food, privacy, waiting time, basic amenities) and the overall atmosphere (or climate) [1], has been recognized as a significant factor influencing the experiences of patients with severe mental illness (SMI) [2-5]. It is an important factor in the quality of patient care [2,6-8], contributing to improved patient satisfaction [9] and improved health outcomes [10,11]. In a recent study, patients identified a welcoming environment as one of the most important aspects of their care [12]. Indeed, a calm and welcoming environment helps to improve patients’ sense of control and empowerment and, consequently, reinforces their willingness to follow recommended treatments. In addition, the care environment is the patients’ first impression and can lead to a positive image of the therapeutic process [13]. A supportive environment promotes communication between patients and staff, can help reduce stressful stimuli, and thus prevents relapses and risky behavior. The priority for psychiatric facilities is therefore to provide patients with a warm and safe atmosphere that allows for positive social interactions, with opportunities for stimulating activities, enabling patients to facilitate their recovery and transition to the community. Different theoretical models can shed light on the additional nonpharmacological and biopsychosocial effects of a patient’s care experience, including the placebo response effects and the set and setting theory [14,15].

Recommended features to promote patient recovery [16-19] include smaller, home-like units with well-decorated common spaces, open designs, access to nature and daylight, and an environment that is clean, well laid out, and ensures privacy and security for personal effects. However, psychiatric facilities are often criticized for prioritizing staff workflow over patient needs [2], leading in some cases to a perceived “prison-like atmosphere” [16,20,21] characterized by conflicting routines and rules and a lack of stimulation [22,23]. Some patients have reported feelings of boredom, loneliness, and stigmatization in these environments [21-26]. The lack of stimulating activities and positive social interactions is a barrier to patients’ successful recovery [24-30]. These negative experiences can contribute to decreased patient satisfaction, increased levels of anxiety and stress among patients, ineffective care, and signs of burnout among staff [27,31,32]. Emphasis should be placed on the design of psychiatric facilities, as a difficult environment is a barrier to care, and patients often perceive such an environment as a lack of attention from staff [30]. In psychiatry, patients cope with an unfamiliar and potentially stressful environment [33], and a better understanding of their experiences is essential to identify and improve current barriers.

Given this growing interest, it is necessary to provide a valid and reliable instrument for measuring patients’ experience of the care environment, applicable to both inpatient and outpatient settings, as care pathways for patients with SMI often combine several care modalities. Previous research has demonstrated that patients with SMI can provide reliable and valid responses to self-administered questionnaires; the impact of psychiatric symptoms and cognitive deficits seems to be negligible [34,35]. The French group PREMIUM (Patient-Reported Experience Measure for Improving Quality of Care in Mental Health) is developing item banks and computerized adaptive tests (CATs) to improve the systematic use of patient-reported experience measures in mental health care [36]. The use of CATs significantly reduces measurement burden by administering a limited number of items targeted to the respondent’s experience level, aiming to improve measurement accuracy.

The objective of this study was to calibrate an item bank and develop a CAT to assess the care environment experienced by adult patients with SMI. These measures will contribute to the current landscape of patient experience measures by providing a valuable complement to PREMIUM measures and capturing what really matters to patients.

## Methods

### Study Population and Procedure

This is a national, multicenter, cross-sectional study conducted between January 2016 and December 2021. Patients were recruited through in- and outpatient psychiatric settings of a French teaching hospital (Assistance Publique-Hôpitaux de Marseille), the FondaMental Foundation’s expert centers [37], and through an online survey. In mental health settings, stable patients who met the inclusion criteria were identified and approached by a member of their usual care team to invite them to participate in the study. The link to the web survey was distributed through patient associations.

Inclusion criteria were as follows: age older than 18 years and younger than 65 years with a diagnosis of schizophrenia, bipolar disorder, or major depressive disorder (MDD), receiving inpatient or outpatient psychiatric care, and speaking or reading French. Vulnerable persons (ie, pregnant or nursing women, persons under legal protection) or those unable to complete a self-administered questionnaire were not included in the study.

Current recommendations suggest a sample size of 300-500 observations for multiparameter item response theory (IRT) models [38-40]. Consequently, we estimated that a sample of around 500 patients would be sufficient to obtain reasonably stable estimates.

### Data Collection

Data were collected through paper questionnaires in health care settings and online through a web survey. Patients reported the following sociodemographic and clinical characteristics: gender, age, educational level, marital status, occupational status, main diagnosis (schizophrenia, bipolar disorders, or MDD), duration of illness, and quality of life (QoL) as measured using the medical outcome study 12-item Short Form (SF-12) [41], which describes 8 QoL dimensions: physical functioning, social functioning, role physical, role emotional, mental health, vitality, bodily pain, general health, and 2 composite scores for physical and mental QoL (ranging from 0 to 100, with higher scores indicating better QoL). Adequate psychometric properties of the SF-12 have been demonstrated among individuals with SMI [42], and the SF-12 has proven to be a good alternative to the SF-36 for minimizing response burden.

The PREMIUM for Care Environment (PREMIUM-CE) item bank consists of 16 items designed for patients with SMI and measures their experience regarding the care environment over the past 4 weeks. Participants respond to the items on a 5-point Likert scale ranging from “strongly disagree” to “strongly agree” with a “not applicable” response option. Additionally, an overall satisfaction item (“Overall, are you satisfied with the health care facilities in which you receive care?”) and a visual analog scale (VAS; minimum 0 to maximum 10) were collected. PREMIUM-CE items were identified through face-to-face interviews with patients with SMI and a systematic review of existing patient-reported experience measure; then the item pool was refined based on an expert review and cognitive interviews with patients with SMI [4,5,36].

### Statistical Analysis

#### Basic Descriptive Analysis

Descriptive statistics were calculated to describe participants’ characteristics, including frequencies and percentages for categorical variables and means and SDs for continuous variables. Response rates, means and SDs, and ceiling and floor effects were also calculated for each item.

#### IRT Assumptions

Unidimensionality, local independence, and monotonicity are the 3 fundamental assumptions of IRT [43]. Data were randomly divided into 2 data sets (n=249 each), one for exploratory factor analysis and one for confirmatory factor analysis (CFA) with the weighted least squares mean and variance estimator to ensure that the PREMIUM-CE was sufficiently unidimensional [44]. Local independence was examined using residual correlations from the final CFA model. Monotonicity was examined using visual inspection of characteristic item curves.

#### Calibration and Fitting an IRT Model

Item parameters were estimated using the generalized partial credit model (GPCM) [45] and compared to the partial credit model [46]. IRT handles missing values by using full information maximum likelihood estimation, which uses all available information, and GPCM is recommended when the amount of missing data is high (20% or more) [38]. Item fit was assessed by examining the mean square infit statistics, which reflect the information-weighted mean squared residuals between the observed and expected response patterns. PREMIUM-CE scores (θ) were estimated by the Bayesian Expected a Posteriori estimation method [47], and a linear transformation was performed to obtain PREMIUM-CE scores ranging from 0 to 100 (higher scores indicate better experience with the care environment). The information curve of the final item bank was calculated, and high measurement precision was defined as an information score >10, corresponding to a reliability of >0.90 [39].

#### Differential Item Functioning Analysis

DIF was examined using ordinal logistic regression models [48,49] by gender (man vs woman), age (median distribution), care setting (outpatient vs inpatient), psychiatric diagnosis (schizophrenia vs bipolar disorder vs MDD), and mode of study participation (online survey vs health care settings).

#### External Validity

Construct validity was examined through convergent and discriminative validity assessments. For convergent validity, Spearman’s rank correlations were computed between PREMIUM-CE scores and both satisfaction (global satisfaction item and VAS) and QoL (SF-12 subscales and composite scores) scores. Our hypothesis was that PREMIUM-CE scores would have strong correlations with satisfaction scores (*r*>0.60), which are 2 related measures, and weak correlations with QoL scores (*r*<0.30). For discriminant validity, relationships between PREMIUM-CE scores and sociodemographic and clinical characteristics of the respondents were examined by using 2-tailed *t* tests, ANOVA, and Pearson correlations. The Q-Q plot was used to determine that the data are approximately normally distributed. Based on previous studies of the determinants of patient satisfaction with psychiatric services [3,50,51], our hypotheses were that higher levels of patient experience of the care environment were associated with older age, being female, being nonsingle, or being in an outpatient setting.

#### CAT Simulations

These simulations using participants’ actual responses were run from the calibrated item bank and compared to identify the best performing CAT version. The stopping rules were based on standard error of measurement (SEM) values of 0.33, 0.44, and 0.55 (corresponding to a reliability between 0.90 and 0.70 [52]). The item administered at baseline was the one that offered the most information to the population mean (θ=0), and then items were administered according to the maximum Fisher information criterion [53].

The indicators used at each stage of the psychometric analyses are presented in Table S1 in Multimedia Appendix 1 [44,54-70]. All of the statistical analyses were performed using the following software: SPSS (version 20.0; IBM Corp), MPlus (version 7.0; Muthen & Muthen), and R (version 4.2.0; R Core Team), using packages *mirt* [71], *lordif* [72], *BifactorIndicesCalculator* [73], and *mirtCAT* [74]. A 2-tailed *P*<.05 was considered statistically significant.

### Ethical Considerations

This study was conducted in accordance with the Declaration of Helsinki and approved by the relevant ethics committee (2014-A01152-45). The study was registered in ClinicalTrials.gov (NCT02491866). All participants provided nonopposition, as required by French law. Additionally, all data were anonymized.

## Results

### Sample Characteristics

Of the 498 participants, 50.2% (250/498) were men, 72.3% (345/477) were unemployed, 73.5% (350/476) were single, and 70.5% (337/478) had an education level of a bachelor’s degree or higher. The average age was 40.9 (SD 11.9) years, and the mean duration of illness was 12.9 (SD 9.3) years. In total, 51.8% (253/488) of the participants had a diagnosis of schizophrenia, 24.4% (119/488) had bipolar disorder, or 23.8% (116/488) had MDD, and 77.7% (387/497) of them were outpatients. The characteristics of the sample are presented in Table 1.

**Table 1:** Sample characteristics.

Characteristics				Values
**Study participation, n/N (%)**				
	Health care setting			271/498 (54.4)
	Online survey			227/498 (45.6)
**Sociodemographic data**				
	Gender (man), n/N (%)		250/498 (50.2)	
	Age (years), mean (SD; n=496)		40.9 (11.9)	
	Marital status (single), n/N (%)		350/476 (73.5)	
	Educational level (<bachelor’s degree), n/N (%)		141/478 (29.5)	
	Employment status (unemployed), n/N (%)		345/477 (72.3)	
**Clinical data**				
	**Care setting, n/N(%)**			
		Outpatient	387/498 (77.7)	
		Inpatient	111/498 (22.3)	
		Inpatient with involuntary commitment	40/111 (36.1)	
	**Main diagnosis (n=488), n (%)**			
		Schizophrenia	253 (51.8)	
		Bipolar disorder	119 (24.4)	
		Major depressive disorder	116 (23.8)	
	**Duration of illness (years; n=469)**			
		Value, mean (SD)	12.9 (9.3)	
		<5 years, n (%)	105 (22.4)	
		≥5 years, n (%)	364 (77.6)	
	**Quality of life (SF-12^a^ scores), mean (SD)**			
		Physical functioning (n=490)	46.5 (11.4)	
		Social functioning (n=491)	34.3 (11.8)	
		Role physical (n=491)	40.5 (11.1)	
		Role emotional (n=491)	33.3 (12.4)	
		Mental health (n=493)	45.0 (11.1)	
		Vitality (n=491)	51.2 (10.3)	
		Bodily pain (n=493)	44.1 (12.8)	
		General health (n=492)	34.8 (10.5)	
		Physical composite (n=484)	43.8 (10.3)	
		Mental composite (n=484)	39.3 (11.5)	

^a^SF-12: 12-item Short Form.

### Basic Descriptive Statistics

The mean item scores ranged from 2.07 (SD 1.32) to 3.24 (SD 0.89), and most items had a missing data rate <10% (except items CE10, CE12, and CE15). The floor and ceiling effects ranged from 1.8% to 10.6% and from 10% to 45.2%, respectively. The interitem correlation values ranged from 0.01 to 0.79, and 3 pairs of items showed too high interitem correlations (>0.70): items CE3-CE4 (*r*=0.73), items CE3-CE5 (*r*=0.78), and items CE4-CE5 (*r*=0.79). Items CE3 and CE5 were excluded because their content was considered less relevant than the remaining items. The lowest scores were for item CE15 (“food was of good quality”), item CE12 (“you had access to media (telephone, computer, internet or Wi-Fi connection, etc),” and item CE10 (“the health care facilities were well equipped”). Table 2 summarizes the distribution of responses for each item.

**Table 2:** Descriptive statistics of PREMIUM-CE item bank.

Item number	Content item	Score, mean (SD)	Floor effect (%)	Ceiling effect (%)	Missing values (%)	Skewness coefficient	Interitem correlations (Range)
CE1	The health care facilities were easily accessible (distance from home, parking, etc)	3.13 (1.09)	4.6	45.2	2	–1.42	0.01-0.66
CE2	The health care facilities were easy to find (eg, signage present and adapted)	3.13 (1.04)	3.4	43.4	1.8	–1.34	0.10-0.51
CE3	The health care facilities were welcoming	2.86 (1.17)	6.2	35.1	0.2	–0.96	0.27-0.78
CE4	The health care facilities were well-laid-out	2.91 (1.09)	4.8	33.5	0.4	–1.04	0.26-0.79
CE5	The health care facilities were pleasant	2.68 (1.19)	6.8	28.7	0	–0.72	0.27-0.79
CE6	The health care facilities were quiet enough	2.83 (1.15)	7.0	30.9	0.4	–1.06	0.21-0.50
CE7	The health care facilities were comfortable (chairs, armchairs, beds, etc)	2.89 (1.07)	4.8	30.1	0.4	–1.09	0.31-0.64
CE8	The health care facilities were clean	3.24 (0.89)	1.8	44.8	0.2	–1.46	0.21-0.63
CE9	The health care facilities were adapted to your needs	3.01 (1.03)	3.4	36.3	1.2	–1.13	0.23-0.68
CE10	The health care facilities were well equipped (materials for activities, group rooms, etc)	2.63 (1.22)	5.8	21.3	20.5	–0.68	0.25-0.66
CE11	The waiting time was acceptable	2.76 (1.21)	7.2	30.7	1.8	–0.90	0.30-0.49
CE12	You had access to media (telephone, computer, internet or Wi-Fi connection, etc)	2.24 (1.39)	10.0	17.5	26.7	–0.21	0.18-0.52
CE13	The sanitary facilities (toilets, bathroom, etc) were clean	3.08 (1.05)	3.2	39.2	7.4	–1.24	0.19-0.63
CE14	The health care facilities guarantee the respect for your privacy	3.12 (1.06)	4.4	41.8	4.6	–1.41	0.25-0.59
CE15	The food was of good quality, if you had to eat	2.07 (1.32)	10.6	10.0	39.4	–0.15	0.01-0.31
CE16	The smoking ban was respected	3.16 (1.02)	3.2	42.6	6.8	–1.37	0.21-0.50

### IRT Assumptions

In EFA, 2 factors had eigenvalue greater than 1, and the scree plot and parallel analysis indicated 2 factors. The eigenvalue of the first factor was 6.46 and explained 46.11% of the total variance; the second factor was 1.33, and the ratio was 4.86. Evaluations indicated that the 2 spatial accessibility items (CE1 and CE2) may form a separate factor, and after a content review, only item CE1 was kept as it was deemed the most relevant. The 1-factor CFA model provided evidence to support the unidimensionality of the remaining 13 items (root mean square error of approximation [RMSEA]=0.082; 95% CI 0.067-0.097; comparative fit index=0.974; Tucker-Lewis index=0.968) and no items showed local dependence (all residual correlations were above |0.20|). Of the 13 items in the bank, 10 were recoded to meet the monotonicity assumption (Table S2 in Multimedia Appendix 1), which improved the model fit (Akaike information criterion=–3343.78 and Bayes information criterion=–3428). Cronbach α was .91.

### Calibration and Fitting an IRT Model

**Figure 1:**
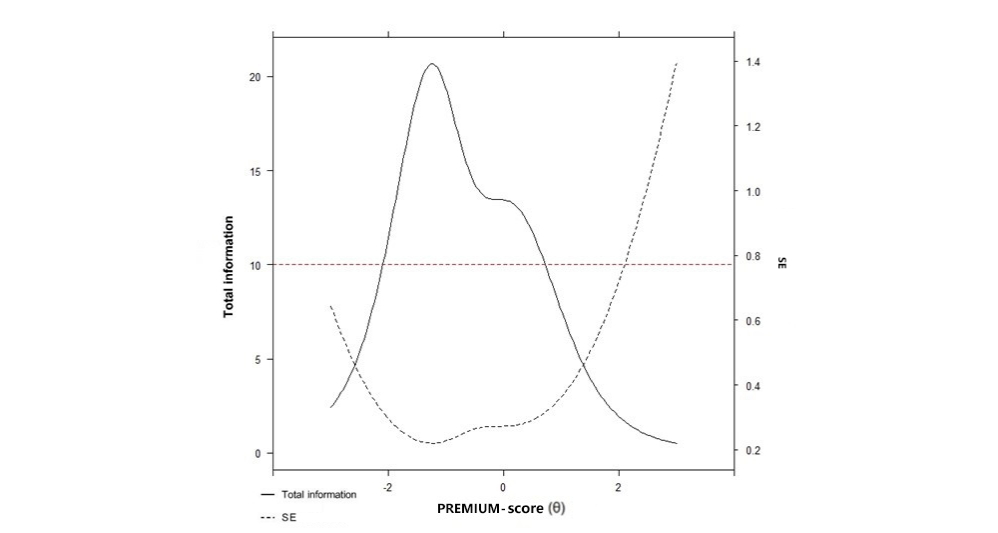
The test information for the Patient-Reported Experience Measure for Improving Quality of Care in Mental Health for Care Environment (PREMIUM-CE) item bank.

The GPCM was used to calibrate the item bank and showed superior fit compared to the partial credit model (10,192.60 and 10,367.43 for Akaike information criterion and 10,382.07 and 10,506.38 for Bayes information criterion; and χ^2^=198.84; *P*<.001); item fit was good (infit values ranging between 0.74 and 1.00). IRT parameter estimates for the 13 items showed slopes ranging from 0.55 to 2.85 and thresholds ranging from –2.07 to 2.29. Item parameters and item fit are provided in Table S2 in Multimedia Appendix 1. As shown in Figure 1, PREMIUM-CE provided the most information in the scale range between –2.6 and 1.4 and had a high measurement accuracy (reliability >0.90) in a shorter range between –2.1 and 0.7 (which corresponds to 88.6% of total information). Item CE7 was the most informative of the bank—“the health care facilities were comfortable,” whereas item CE15 was the least informative—“the food was of good quality.”

### Differential Item Functioning Analysis

Responses to items CE6 (quiet) and CE13 (sanitary) were flagged for overall DIF but with negligible magnitude according to health care settings. Likewise, the DIF magnitude was negligible for item CE16 (smoking ban) according to mode of study participation and for item CE15 (food) according to gender, mode of study participation, and diagnostic after pooling bipolar disorder and MDD (mood disorders vs schizophrenia; *P*=.02; Δ*R*^2^=.013). None of the items showed significant DIF for age. DIF results are provided in Table S3 in Multimedia Appendix 1.

### External Validity

As expected, there were strong correlations between the PREMIUM-CE item bank and overall satisfaction and VAS, supporting convergent validity. Similarly, all SF-12 dimensions were weakly correlated with the PREMIUM-CE item bank, except for bodily pain and vitality. Associations were found between better experience of the care environment (ie, higher PREMIUM-CE scores) and older age, being a woman, being voluntarily admitted to a hospital, and being recruited through health care facilities. There was no significant effect of educational level, marital status, employment status, diagnosis, or duration of illness. These results are presented in Table 3.

**Table 3:** Comparison of PREMIUM-CE scores with sociodemographic and clinical data and proxy measures of quality of care.

Characteristics				Correlation coefficient (*r*)		Mean (SD)		*P* value	
**Study participation**									<.001
	Health care setting			N/A^a^		63.12 (17.58)			
	Online survey			N/A		52.53 (20.34)			
**Sociodemographic data**									
	Age			0.19		N/A		<.001	
	**Gender**								.04
		Man	N/A		56.52 (18.84)				
		Woman	N/A		60.18 (20.19)				
	**Marital status**								.51
		Single	N/A		58.67 (19.12)				
		Nonsingle	N/A		57.33 (21.37)				
	**Educational level**								.07
		<Bachelor’s degree	N/A		60.88 (18.86)				
		≥Bachelor’s degree	N/A		57.34 (20.04)				
	**Employment status**								.60
		Employed	N/A		57.58 (19.36)				
		Unemployed	N/A		58.64 (19.95)				
**Clinical data**									
	**Care setting**								.01
		Outpatient	N/A		57.34 (20.11)				
		Inpatient voluntarily admitted	N/A		65.54 (17.61)				
		Inpatient involuntarily admitted	N/A		54.33 (14.52)				
	**Main diagnosis**								.58
		Schizophrenia	N/A		57.46 (18.48)				
		Bipolar disorder	N/A		59.73 (20.72)				
		Major depressive disorder	N/A		58.22 (20.97)				
	**Duration of illness**								.14
		<5 years	N/A		60.86 (18.79)				
		≥5 years	N/A		57.64 (20.08)				
**Proxy measures**									
	Item of overall satisfaction			0.78		N/A		<.001	
	Visual analog scale			0.69		N/A		<.001	
	**Quality of life (SF-12^b^ scores)**								
		Physical functioning	0.14		N/A		.003		
		Social functioning	0.19		N/A		<.001		
		Role physical	0.21		N/A		<.001		
		Role emotional	0.19		N/A		<.001		
		Mental health	0.12		N/A		.01		
		Vitality	–0.04		N/A		.41		
		Bodily pain	0.09		N/A		.05		
		General health	0.13		N/A		.004		
		Physical composite	0.14		N/A		.001		
		Mental composite	0.11		N/A		.01		

^a^N/A: not applicable.

^b^SF-12: 12- items short form.

### CAT Simulations

As reported in Table 4, the results of the CAT simulations based on SEM <.33 and <.44 were both acceptable in terms of accuracy and precision, but the scenario based on SEM <.33 (corresponding to a reliability of 0.90) was the most efficient. Of the 498 participants included in the simulation, 79.5% (396) achieved a reliable score with an average of 7 items administered.

**Table 4:** Mean scores and precision indicators for each computerized adaptive test simulation.

Precision level and indicators			Values
**SEM^a^<0.33**			
	Mean (SD)	58.30 (19.39)	
	Correlation coefficient (*r*)	0.98	
	RMSE^b^	0.17	
	Mean number of items	6.95	
**SEM<0.44**			
	Mean (SD)	58.35 (18.75)	
	Correlation coefficient (*r*)	0.95	
	RMSE	0.29	
	Mean number of items	4.46	
**SEM<0.55**			
	Mean (SD)	50.57 (21.27)	
	Correlation coefficient (*r*)	0.92	
	RMSE	0.37	
	Mean number of items	3.10	

^a^SEM: standard error of measurement.

^b^RMSE: root mean square error.

## Discussion

### Principal Findings

In this study, we report the calibration and initial evaluation of a new PREMIUM-CE item bank measuring patients’ experience of the care environment that can be used for CATs. The PREMIUM-CE questionnaire is the first available questionnaire thus far to assess the quality of the care environment, applicable in outpatient and inpatient settings, for adults with SMI. This new measure covers different facets of the care environment, including ease of access in time and space, facility layout and basic amenities, food quality, comfort and cleanliness, respect for privacy, and smoking ban. PREMIUM-CE items address both concerns common to all patients (eg, cleanliness or food) and those more specific to psychiatric patients (eg, therapeutic workshops). Existing instruments measure more objective aspects (eg, checklists fulfilled by direct observation), and patients with SMI were not involved in the development and validation process [75].

PREMIUM-CE has undergone rigorous psychometric evaluation, consistent with previous studies conducted as part of the French PREMIUM initiative [36]. Although the RMSEA was slightly above the criterion of <.08, our results provide evidence of sufficient unidimensionality, and the item pool meets the assumptions for IRT modeling. Research has shown that the RMSEA statistic is problematic for assessing the unidimensionality of item banks measuring health concepts [76], as RMSEA is sensitive to model complexity (number of estimated parameters) and skewed data distributions [77]. These results are comparable to other calibration studies of item banks of patient-reported measures [78-83]. Overall, our results demonstrate that PREMIUM-CE has strong psychometric properties for patients with SMI, with negligible measurement bias by gender, health care settings, and mode of study participation. Items CE10, CE12, and CE15 had a higher rate of missing data than the other items, but this rate was below 40%, which remains acceptable by psychometrics standards [84]. In addition, these items had lower scores compared to others, meaning that efforts should be targeted on these aspects to improve the experience of patients with SMI. Future studies should examine whether changes to these items are required. The absence of a large DIF magnitude according to health care settings will make it possible to study changes in the experience of psychiatric patients over time, for whom care pathways often combine inpatient and outpatient care modalities. The 13 items in the final version of the PREMIUM-CE are listed in Multimedia Appendix 1, Table S4. In addition, the CAT version showed comparable measurement accuracy to the full item bank with high correlations between scores with an average of only 7 items administered.

External validity, explored using validated questionnaires and sociodemographic and clinical data, generally supported our initial hypotheses. Previous research has demonstrated that some factors, such as age, gender, marital status, and physical and mental health status, can influence individuals’ experiences within a specific environment [2,3]. It is important to note, however, that the literature has not consistently established clear associations for age, gender, and marital status [3]. According to our results, older age, being female, being voluntarily admitted, and reporting a good physical and mental quality of life are associated with higher levels of patient experience of the care environment. As previously described [85], women reported higher levels of experience than men. Likewise, older people tend to be more accommodating, perhaps because they have fewer expectations than younger people [86]. Also, contrary to what might be expected, voluntarily admitted patients reported higher levels of experience than outpatients, although some patients reported a preference for community mental health treatment, which they considered less stigmatizing [87] and compatible with professional and social functioning. Furthermore, the literature has shown that hospitalization, particularly in the context of involuntary admission, can have a negative impact on patient experience [3], because it can be experienced as traumatic or particularly stressful for patients [88].

However, our results suggest that patients voluntarily admitted to the hospital may have a more holistic and structured experience compared to outpatients, conducive to positive therapeutic relationships with staff, whereas constraint has a negative effect on therapeutic relationships in the case of involuntarily admitted patients [88-90]. Finally, a positive but weak association was found between higher levels of patient experience and better QoL, as previously reported in other studies [51]. A calm and welcoming care environment contributes to patients feeling more comfortable and safer [50], which can reduce stress and anxiety and enhance relationships with staff, thereby promoting patients’ recovery. Participants completing the online survey reported a poorer experience of the care environment than participants in health care settings because the latter may be more favorable due to fear of a negative effect on their relationships with staff, or this difference may be due to a possible recall bias.

The most poorly rated items by patients were related to accessing equipment (CE10), media (CE12), and food (CE15). Difficulties with access to equipment (eg, for art therapy) and media (eg, televisions or computers) are related to boredom, isolation, frustration, and higher levels of distress in patients [25]. A variety of individual or group activities could be offered to patients, such as therapeutic workshops in self-expression (ie, writing), art (ie, photography or painting), psychosocial rehabilitation (ie, cooking, which may also improve diet habits), or body awareness (ie, sophrology), to help patients develop social skills and promote social reintegration, improve confidence and self-esteem, build emotional resilience, and enjoy themselves. Facilities should have basic amenities such as affordable Wi-Fi and a working television in a common room accessible to all patients. Likewise, rooms should be equipped with a minimum package of free channels; however, not all facilities are equally equipped, and the cost of access to Wi-Fi and pay television channels can vary by as much as 2-fold. The content of what is broadcast on television should also be a therapeutic consideration. For example, it seems logical to avoid broadcasting distressing news or uninspiring programs and to favor the broadcasting of cultural works that could be the object of an exchange after viewing, such as a film club. The use of cell phones in health care settings presents challenges in terms of the potential risk of theft or breakage, as well as concerns about maintaining confidentiality. Additionally, it can be a source of tension with staff (eg, if the telephone credit is exceeded). There is no law that prohibiting the use of cell phones because communicating is a fundamental individual freedom, but the internal rules of the facilities can regulate their use by specifying the times and places of use and prohibit taking pictures of patients and staff. Furthermore, psychiatrists may occasionally prohibit a patient from keeping a cell phone, computer, or tablet as part of a medical decision, particularly in the case of placement in a seclusion room or for medical conditions. Previous studies have shown that a healthy diet is essential for good mental health and can prevent the worsening of symptoms [91,92], and that patients’ satisfaction with hospital food services strongly influences their overall satisfaction with hospital care [93]. Diets such as the Mediterranean diet have been shown to improve patient outcomes [91]. Providing a menu tailored to patient preferences while focusing on food quality (taste, presentation, flavor, preparation, and variety), as well as the hospital environment, will help improve inpatient appetite and satisfaction [93]. In summary, the current challenges of hospital food service are to transition to a diet that is lower in meat, closer to the Mediterranean diet, without plastic packaging, and low in processed products while increasing the attractiveness of local and seasonal products, all while maintaining costs [91]. By contrast, the most highly rated items by patients were related to spatial accessibility (CE1), cleanliness (CE8), and smoking ban (CE16). Although health care facilities are under a total smoking ban throughout their whole facilities (including in specifically dedicated “smoking areas” or outside), the reality is often more flexible to accommodate patients who cannot leave the health care facilities, even temporarily (eg, patients under constraint). Proposals for smoking cessation assistance should be systematically offered to patients.

### Limitations

Some limitations of this study are worth noting. Our sample size, while relatively modest, was sufficient to obtain accurate estimates. Current recommendations suggest that at least 300 observations are sufficient when using multiparameter models like the GPCM [38-40]. However, our results showed that the assumptions required for IRT calibration were met and that the model fit was adequate. In addition, some DIF analyses comparing subgroups with sample sizes smaller than those recommended for DIF analyses (at least 200 observations per group [94]) may have lacked the statistical power to detect a statistically significant DIF. These DIF findings should be regarded as preliminary, and future work with a larger sample will allow us to confirm these results. Although participants from the online survey and those from health care settings may have reported different levels of experience, this mixed survey design was chosen to ensure inclusivity across various subgroups, as supported by previous research on the equivalence of administration methods [95]. DIF analysis revealed that none of the items was flagged with a large DIF magnitude according to the patient’s mode of study participation, suggesting that the data can be pooled without substantial bias. It was not possible to calculate a participation rate or to compare the characteristics of respondents and nonrespondents. This study was widely disseminated nationally, and our sample included inpatients and outpatients with diverse characteristics from different geographic regions of the country. Patients self-reported their diagnosis, and some data (488/498, 2.5%) were missing. However, the risk of misdiagnosis is considered minimal because all participants were fully informed about the study scope and diagnostic criteria. Additionally, this approach closely mirrors the real-world conditions of PREMIUM use. The title of the study mentioned general experience of care to limit the self-selection bias of patients with extreme care environment experiences. Future work will confirm the generalizability of our results. PREMIUM-CE has greater measurement accuracy for patients with scores between –2.1 and +0.7 (ie, reporting low to moderate levels of experience), and thus more items are needed to estimate scores for patients at both ends of the latent continuum. Future work should also reevaluate the precision and accuracy of the CAT in an independent sample and under real-world conditions. Finally, criterion validity could not be assessed because, to our knowledge, no gold standard was available and evidence for construct validity was limited. Future validation studies should examine the relationship between this new measure and objective assessments of the care environment (eg, evaluation by architects or other professionals).

### Conclusion

The PREMIUM-CE item bank and its CAT version have demonstrated strong psychometric properties, making them robust measures for assessing patient experience of the care environment, applicable in both outpatient and inpatient settings, for adults with SMI. These measures contribute to the current landscape of patient experience measures by providing a valuable complement to PREMIUM measures of what really matters to patients.

## Appendix

Multimedia Appendix 1 Indicators of psychometric performance, parameter estimates (discrimination and thresholds) and fit statistics, differential item functioning results, and list of the 13-item of the Patient-Reported Experience Measure for Improving Quality of Care in Mental Health for Care Environment (PREMIUM-CE) item bank (English and French versions).

## Abbreviations

CAT: computerized adaptive test
CFA: confirmatory factor analysis
DIF: differential item functioning
GPCM: generalized partial credit model
IRT: item response theory
MDD: major depressive disorder
PREMIUM: Patient-Reported Experience Measure for Improving Quality of Care in Mental Health
PREMIUM-CE: Patient-Reported Experience Measure for Improving Quality of Care in Mental Health for Care Environment
QoL: quality of life
RMSEA: root mean square error of approximation
SEM: standard error of measurement
SF-12: 12-item Short Form
SMI: severe mental illness
VAS: visual analog scale
