# Supplementary material for: Psychometric Assessment of an Item Bank for Adaptive Testing on Patient-Reported Experience of Care Environment for Severe Mental Illness: Validation Study
Source: JMIR Ment Health. 2024 May 16;11:e49916. doi: 10.2196/49916 (PMC11140279; doi:10.2196/49916)
Supplement: Multimedia Appendix 1 [file mental_v11i1e49916_app1.docx]

**Table S1**. Indicators of psychometric performance.

| **Statistical analyses stage** | | **Indicators** |
| --- | --- | --- |
| Basic descriptive analysis | | Items are deleted if at least one of the following conditions is met:  - high amount of missing data at item-level (>70%) [68]  - skewness (≤95% of response rate in one category or an absolute coefficient ≤4) [69]  - high inter-item correlation coefficients (> 0.70) [68,70]  - Cronbach’s alpha coefficient (α > 0.70) [54] |
| **IRT assumptions** | | |
|  | Unidimensionality | - The acceptable fit of a 1-factor CFA model was evaluated according to the following criteria: RMSEA < 0.08 / CFI > 0.95 / TLI > 0.95 [55,56]  - The underlying structure of the item bank was examined in EFA according to the following criteria: Kaiser-Guttman rule (eigenvalues ≥ 1), scree test, parallel analysis, and factor loadings (≥ 0.30). To support sufficient unidimensionality, a difference in magnitude between the first and second eigenvalues (ratio > 4) and at least 20% of the variability explained by the first factor [44] were expected |
|  | Local independence | - Residual correlations matrix of the 1-factor CFA model ≤.25 [57,58] |
|  | Monotonicity | - Item characteristics curves (ICC) were recoded based on deviations in AIC and BIC criteria between the final model (with recoded items) and the initial model (without recoded items) [59,60] |
| Calibration and fitting to an IRT model | | - Comparison with PCM evaluated by: likelihood ratio test (X^2^)[ 61], AIC and BIC criteria [59,60]  - Discrimination parameter ≥0.50 [62,63]  - Item fit was evaluated according to infit values between the range [0.7-1.3] [64,65] |
| Differential item functioning analysis | | For each item, an overall test for both uniform and non-uniform DIF at *P*<.01 was performed and the DIF magnitude was evaluated by the pseudo McFadden’s R^2^ (ΔR^2^) according to Zumbo's classification: negligible if ΔR^2^ < 0.13, moderate if 0.13 < ΔR^2^ < 0.26 and large if ΔR^2^ > 0.26 [66] |
| CAT simulations | | - Accuracy: correlation coefficients (r) between CAT scores and scores for the whole item bank ≥ 0.90  - Precision: RMSE ≤ 0.30 [67] |

**Abbreviations:** *IRT* item response theory / *CFA* confirmatory factor analysis / *RMSEA* root mean square error of approximation / *CFI* comparative fit index / *TLI* Tucker–Lewis index / *EFA* exploratory factor analysis / *PCM* partial credit model / *AIC* Akaike information criterion / *BIC* Bayes information criterion / *DIF* differential item functioning / *RMSE* root mean square error / *CAT* computerized adaptive testing.

**Table S2**. Parameter estimates (discrimination and thresholds) and fit statistics for the 13 items in the final PREMIUM-CE item bank.

| **Item number** | **Discrimination** | **Threshold 1** | **Threshold 2** | **Threshold 3** | **Threshold 4** | **Infit** |
| --- | --- | --- | --- | --- | --- | --- |
| **CE1^a^** | 1.06 | –1.89 | 0.01 | — | — | 0.92 |
| **CE4** | 2.09 | –1.58 | –1.19 | –0.94 | 0.43 | 0.81 |
| **CE6^a^** | 1.76 | –1.39 | 0.61 | — | — | 1.00 |
| **CE7** | 2.18 | –1.57 | –1.16 | –1.01 | 0.58 | 0.74 |
| **CE8^a^** | 2.85 | –1.74 | 0.07 | — | — | 0.84 |
| **CE9** | 1.90 | –1.80 | –1.32 | –1.10 | 0.33 | 0.91 |
| **CE10^a^** | 2.42 | –1.05 | 0.60 | — | — | 0.94 |
| **CE11^a^** | 1.24 | –1.35 | 0.67 | — | — | 0.87 |
| **CE12^a^** | 1.17 | –0.53 | 0.75 | — | — | 0.96 |
| **CE13^a^** | 1.57 | –1.72 | 0.19 | — | — | 0.90 |
| **CE14^a^** | 2.72 | –1.45 | 0.10 | — | — | 0.81 |
| **CE15^a^** | 0.55 | –1.09 | 2.29 | — | — | 0.91 |
| **CE16^a^** | 1.22 | –2.07 | 0.08 | — | — | 0.91 |

^a^items recoded to meet the monotonicity assumption.

**Table S3.** Differential item functioning (DIF) results.

| **Item number** | **Sex** | | **Age** | | **Care setting** | | **Main diagnosis** | | **Study participation** | |
| --- | --- | --- | --- | --- | --- | --- | --- | --- | --- | --- |
|  | **P value** | **ΔR²** | **P value** | **ΔR²** | **P value** | **ΔR²** | **P value** | **ΔR²** | **P value** | **ΔR²** |
| **CE1** | .21 | — | .90 | — | .08 | — | .33 | — | **.004** | **.012** |
| **CE4** | .47 | — | .68 | — | .97 | — | .24 | — | .93 | — |
| **CE6** | .86 | — | .90 | — | .01 | — | .201 | — | .16 | — |
| **CE7** | .15 | — | .92 | — | **.003** | **.010** | .41 | — | .49 | — |
| **CE8** | .53 | — | .73 | — | .06 | — | .30 | — | .10 | — |
| **CE9** | .56 | — | .80 | — | .41 | — | .59 | — | .46 | — |
| **CE10** | .34 | — | .72 | — | .08 | — | .21 | — | .75 | — |
| **CE11** | .73 | — | .77 | — | .89 | — | .89 | — | .90 | — |
| **CE12** | .97 | — | .73 | — | .58 | — | .14 | — | .11 | — |
| **CE13** | .66 | — | .61 | — | **<.001** | **.052** | .39 | — | .93 | — |
| **CE14** | .16 | — | .83 | — | .13 | — | .32 | — | .74 | — |
| **CE15** | **.006** | — | .71 | — | .22 | — | .09 | — | **<.001** | **.034** |
| **CE16** | .93 | — | .18 | — | .03 | — | .52 | — | **.002** | **.014** |

**Notes:** Bold values indicate DIF at *P* value <.01

ΔR²: DIF magnitude: negligible (ΔR² < .13), moderate (.13 ≤ ΔR² ≥ .26), or large (ΔR² ≥ .26)

**Table S4**. List of the 13-item of the PREMIUM-CE item bank (English and French version).

| **Item number** | **Item content in English** | **Item content in French** |
| --- | --- | --- |
|  | **Over the past 4 weeks, you have found that:** | **Au cours des 4 dernières semaines, vous avez constaté que :** |
| **CE1** | the health care facilities were easily accessible (e.g. distance from home, parking, etc.) | les lieux de soins étaient facilement accessibles (ex : distance depuis chez vous, parking, etc.) |
| **CE4** | the health care facilities were well-laid-out | les lieux de soins étaient bien aménagés |
| **CE6** | the health care facilities were quiet enough | les lieux de soins étaient suffisamment silencieux |
| **CE7** | the health care facilities were comfortable (e.g. chairs, armchairs, beds, etc.) | les lieux de soins étaient confortables (ex : chaises, fauteuils, lits, etc.) |
| **CE8** | the health care facilities were clean | les lieux de soins étaient propres |
| **CE9** | the health care facilities were adapted to your needs | les lieux de soins étaient adaptés à vos besoins |
| **CE10** | the health care facilities were well equipped (e.g. materials for activities, group rooms, etc.) | les lieux de soins étaient bien équipés (ex : matériels pour les activités, salles de groupe, etc.) |
| **CE11** | the waiting time was acceptable | le temps d’attente au sein des lieux de soins était acceptable |
| **CE12** | you had access to media (telephone, computer, internet / wifi connection, etc.) | vous avez eu accès à des médias (téléphone, ordinateur, connexion internet / wifi, etc.) |
| **CE13** | the sanitary facilities (toilets, bathroom, etc.) were clean | les sanitaires (toilettes, salle de bain, etc.) mis à votre disposition étaient propres |
| **CE14** | the health care facilities guarantee the respect of your privacy | les lieux de soins garantissaient le respect de votre intimité |
| **CE15** | the food was of good quality, if you had to eat | la nourriture était de bonne qualité, si vous avez été amené(e) à manger sur vos lieux de soins |
| **CE16** | the smoking ban was respected | l’interdiction de fumer dans les locaux était respectée |
